# Unusual complications and corneal clearance after Descemet Membrane Endothelial Transfer in Pseudophakic Bullous Keratopathy

**Published:** 2019

**Authors:** Anil Rahul Rachwani, Carlos Rocha-de-Lossada, Calvo de Mora Marina Rodríguez, Baca Vaca Gustavo Fernández

**Affiliations:** * Ophthalmology Department, Hospital Regional Universitario de Málaga, Spain

**Keywords:** malignant glaucoma, DMEK, DMET, bullous keratopathy, infectious crystalline keratopathy

## Abstract

**Purpose.** to report malignant glaucoma and infectious crystalline keratopathy as complications after an uneventful Descemet Membrane Endothelial Keratoplasty (DMEK), and corneal clearance despite graft detachment after the surgery in a patient with pseudophakic bullous keratopathy.

**Method.** A 81-year-old patient with high Intraocular Pressure (IOP) and flat anterior chamber with patent iridotomies after DMEK was diagnosed of malignant glaucoma. The medical approach being insufficient, the patient required a pars-plana vitrectomy, capsulo-hyaloidectomy, and surgical iridectomy.

**Results.** The IOP was reduced and anterior chamber was repositioned after surgical management. Corneal clearance was observed despite graft detachment. The patient developed an infectious crystalline keratopathy after the resolution of malignant glaucoma.

**Conclusions.** malignant glaucoma is a rare complication following DMEK. Corneal clearance can be attained despite graft detachment after DMEK probably due to an unintentional Descemet Membrane Endothelial Transfer (DMET). However, in low dosage, steroid treatment remains a risk factor for developing ICK.

**Abbreviations:** PBK = Pseudophakic Bullous Keratopathy, DMEK = Descemet Membrane Endothelial Keratoplasty, DMET = Descemet Membrane Endothelial Transfer, IOP = Intraocular Pressure, BCVA = Best Corrected Visual Acuity, AC = Anterior Chamber, MG = Malignant Glaucoma, ICK = Infectious Crystalline Keratopathy

## Introduction

Pseudophakic Bullous Keratopathy (PBK) is a common indication of corneal transplantation. Descemet Membrane Endothelial Keratoplasty (DMEK) is increasingly performed due to its excellent results, low graft rejection rate and reduced need of steroids [**1**]. Nevertheless, DMEK is not without complications [**2**], graft detachment being the most common complication. Either air or expandable gas is used both in the straightforward surgery and in the “re-bubbling” procedure to treat graft detachment. The main cause of raised Intraocular Pressure (IOP) in the early postoperative period is usually a reverse pupillary block secondary to a full air or gas fill that pushes the iris backwards [**3**,**4**].

## Case report 

An 81-year-old hyperopic patient with pseudoexfoliation syndrome developed PBK after complicated cataract surgery. Her Best Corrected Visual Acuity (BCVA) was 1/ 20 Snellen (1.30 logMAR). The patient underwent an uneventful DMEK surgery, but the afternoon after the procedure the air bubble was located behind the iris, evidencing a flat Anterior Chamber (AC), high IOP (50 mmHg) and negative Seidel test. The inferior iridotomy was patent. Topical atropine, phenylephrine, apraclonidine and timolol, oral 250mg t.i.d. acetazolamide, and intravenous mannitol were prescribed. The next day, AC was still flat, so laser YAG iridotomy enlargement and capsulo-hyaloidectomy was attempted, though it was difficult due to corneal oedema. Subsequently, the air bubble was removed surgically by pars-plana aspiration. Later that evening, the IOP was 45 mmHg and flat AC was persistent. Hence, Malignant Glaucoma (MG) was suspected and the next day a pars-plana vitrectomy with capsulo-hyaloidectomy and iridectomy was performed with successful results. 

Corneal oedema and a graft detachment located inferiorly in the AC were noticed in the early follow-up. Interestingly, corneal oedema decreased progressively and six months after DMEK the patient attained a spontaneous BCVA of 4/ 10 Snellen (0.39 LogMar), central pachymetry of 550 µm and medically controlled glaucoma. The patient received a long-term steroid treatment as per postoperative DMEK protocol and wore a bandage contact lens. One year after the surgery, the patient developed an infectious crystalline keratopathy (ICK). Microbiological cultures proved *Candida glabrata* to be the cause. The patient received topical, intrastromal and oral Amphotericin B, and is currently controlled with topical treatment. The patient does not consent for a further corneal surgical approach.

## Discussion

MG is defined as a uniform flattening of the AC in an eye with raised IOP in the presence of a patent iridotomy. It is thought that the cause is a misdirection of the aqueous humour towards the vitreous cavity, shifting the lens-iris diaphragm forward. MG is described as a complication of most intraocular surgeries, especially glaucoma surgery in patients with prior angle closure, being more frequent in hyperopic eyes [**3**]. A careful differential diagnosis of MG should be undergone. Pupillary block presents a moderate depth of the central AC and iris bombée. Choroidal detachments and effusions are typically hypotonic. Suprachoroidal haemorrhage usually presents severe pain and is often preceded by hypotony [**5**]. Medical approach of MG is recommended during the first five days using mydriatic and cycloplegic agents in order to relax the ciliary muscle and retracting the lens-iris diaphragm. This can also be achieved by dehydrating the vitreous using hyperosmotics. Aqueous suppressants are used to decrease posterior pooling of aqueous humour. In refractory cases, Nd:YAG anterior hyaloidotomy should be considered in pseudophakic and aphakic eyes, allowing movement of fluid between the posterior and anterior segments of the eye. If ineffective, pars plana vitrectomy with capsulo-hyaloidectomy and lensectomy should be considered [**5**]. 

In the present case, MG was caused by air bubble migration to the posterior chamber by an unknown mechanism. It may have been due to an abnormal communication caused by the prior complicated cataract surgery. Another cause of MG might have been the saline irrigation in the AC to unfold the graft that could have accumulated behind the iris triggering aqueous misdirection. YAG iridotomy enlargement and capsulo-hyaloidectomy was attempted but proved difficult due to corneal oedema. As air bubble removal by pars-plana aspiration proved unsuccessful, a pars-plana vitrectomy with capsulo-hyaloidectomy and iridectomy was performed. 

Descemet Membrane Endothelial Transfer (DMET) is performed by injecting the donor graft consisting in Descemet Membrane and endothelium into the anterior chamber after descemetorhexis, without fully unfolding it, but with some contact area between graft and host cornea [**6**,**7**]. In the case we presented, we hypothesized that the irido-corneal contact, in the context of MG after DMEK, caused an almost fully detached graft, hence, the procedure ended up being an unintentional DMET. Interestingly, the corneal oedema improved in the following months. Several cases of corneal oedema resolution in presence of DMEK graft detachment, or even descemetorhexis with no posterior graft transplant have been reported. Dirisamer et al. proposed that contact between donor and host cornea is required to achieve corneal clearance as they reported successful results in patients with partial or large detachments, but not in eyes with a “free-floating graft roll” in the anterior chamber [**6**,**7**]. Our case matched these findings, as the graft was not completely detached. 

Birbal et al. reported unsuccessful long-term results in eight patients receiving DMET. They experienced corneal clearance in eyes with Fuchs endothelial dystrophy (FED) but not PBK, suggesting that the mechanism of DMET might be to stimulate host endothelial migratory response rather than to supply functional cells [**8**]. Shah et al. reported an increase in endothelial population despite the extraction of the graft and in descematorrhexis-alone procedures, suggesting a possible mechanism of host endothelial cell migration [**9**]. Dirisamer also had similar results and hypothesized that the recipient endothelium must be primarily involved due to the difference in clinical outcome of DMET in PBK and FED. Hence, DMET is ineffective in eyes with total deficiency of endothelial cells (PBK), yet if performed after descematorrhexis in patients with a pathologically altered subcellular matrix (FED) results in re-endothelialization and corneal clearance [**9**]. However, in our patient with severe PBK the corneal oedema improvement was evident despite the graft being detached. Although the mechanism is uncertain, we reason that DMET might have contributed to recover partially the corneal transparency [**6**].

One year after the surgery, our patient developed ICK, probably because of the combination of topical corticosteroids and bandage contact lens, as ICK typically occurs after an epithelial defect from a surgical procedure and is potentiated by localized immunosuppression, commonly steroids. Many pathogens can cause ICK although *streptococci* remain the classic aetiology. It is characterized by insidious infiltration of white or grey opacities within the corneal stroma, appearing as stellate or branching opacities. If topical and/ or intrastromal antibiotics/ antifungals are unsuccessful, Nd:YAG laser can be used to disrupt the microbial biofilm. Ultimately, patients will need to undergo keratoplasty [**10**].

Although rare, malignant glaucoma can be a complication after DMEK surgery, and we must suspect it in postoperative patients with high IOP and flat AC in the presence of a patent iridotomy. Although steroid dosage is usually lower after DMEK compared to other keratoplasty techniques, it remains a risk factor for the growth of stromal biofilm that could ultimately lead to ICK, especially if bandage contact lens is worn.

**Fig. 1:**
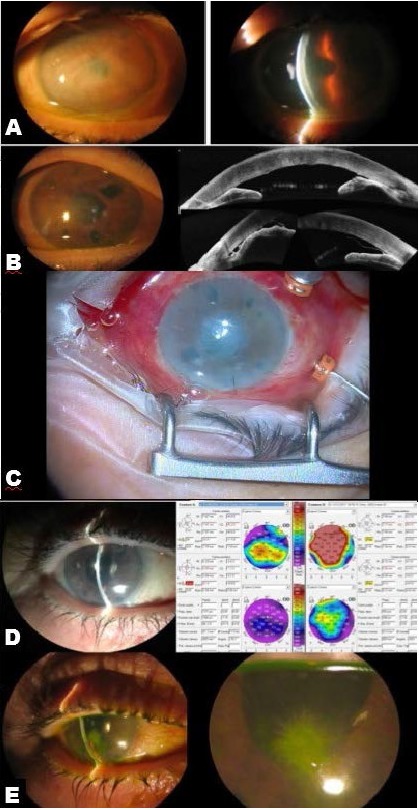
**A.** Preoperative status. Corneal oedema in patient with Bullous Keratopathy. **B.** Immediate Postoperative status. Iridocorneal peripheral adhesions and graft detachment. Notice the enlarged iridotomies. **C.** Intraoperative image of the pars plana vitrectomy and capsulo-hyaloidectomy. Notice the vitretome behind the inferior iridectomy. **D.** Corneal clearance six months after DMEK. **E.** Corneal oedema resolution proved in the pachymetry values. Contact lens on. **F.** One year after DMEK. Infectious crystalline

**Disclosures**

Patient informed consent was obtained.

**Conflict of interest**

Conflicts of interest and source of funding: none.

**Acknowledgements**

None.
